# Curcuminoids Extract, Hydrolyzed Collagen and Green Tea Extract Synergically Inhibit Inflammatory and Catabolic Mediator’s Synthesis by Normal Bovine and Osteoarthritic Human Chondrocytes in Monolayer

**DOI:** 10.1371/journal.pone.0121654

**Published:** 2015-03-23

**Authors:** Fanny Comblain, Christelle Sanchez, Isabelle Lesponne, Marc Balligand, Samuel Serisier, Yves Henrotin

**Affiliations:** 1 Bone and Cartilage Research Unit, Arthropôle Liège, University of Liège, CHU Sart-Tilman, Liège, Belgium; 2 Royal Canin Research Center, Aimargues, France; 3 Department of Clinical Sciences, Faculty of Veterinary Medicine, University of Liège, Liège, Belgium; 4 Physical Therapy and Rehabilitation department, Princess Paola Hospital, Marche-en-Famenne, Belgium; National Institute of Health and Medical Research, FRANCE

## Abstract

The main objective of this study was to assess the *in vitro* effects of curcuminoids extract, hydrolyzed collagen and green tea extract in normal bovine chondrocytes and osteoarthritic human chondrocytes cultured in monolayer. This study also investigated the synergic or additive effects of these compounds. Enzymatically isolated primary bovine or human chondrocytes were cultured in monolayer until confluence and then incubated for 24 hours or 48 hours in the absence or in the presence of interleukin-1β and with or without curcuminoids extract, hydrolyzed collagen or green tea extract, added alone or in combination, at different concentrations. Cell viability was neither affected by these compounds, nor by interleukin 1β. In the absence of interleukin-1β, compounds did not significantly affect bovine chondrocytes metabolism. In human chondrocytes and in the absence of interleukin 1β, curcuminoids extract alone or in combination with hydrolyzed collagen and green tea extract significantly inhibited matrix metalloproteinase-3 production. In interleukin-1β-stimulated bovine chondrocytes, interleukin-6, inducible nitric oxide synthase, cyclooxygenase2, matrix metalloproteinase 3, a disintegrin and metalloproteinase with thrombospondin type I motifs 4 and a disintegrin and metalloproteinase with thrombospondin type I motifs 5 expressions were decreased by curcuminoids extract alone or in combination with hydrolyzed collagen and green tea extract. The combination of the three compounds was significantly more efficient to inhibit interleukin-1β stimulated matrix metalloproteinase-3 expression than curcuminoids extract alone. In interleukin-1β-stimulated human chondrocytes, nitric oxide, interleukin-6 and matrix metalloproteinase 3 productions were significantly reduced by curcuminoids extract alone or in combination with hydrolyzed collagen and green tea extract. These findings indicate that a mixture of curcuminoids extract, hydrolyzed collagen and green tea extract has beneficial effects on chondrocytes culture in inflammatory conditions and provide a preclinical basis for the *in vivo* testing of this mixture.

## Introduction

Osteoarthritis (OA) is a chronic, painful, degenerative and inflammatory condition that affects the joints and leads to functional disability. The most prominent feature of OA is the progressive degradation of articular cartilage. Chondrocytes play a key role in cartilage degradation in OA by producing matrix metalloproteinases (MMP), free radicals and inflammatory cytokines in response to mechanical or biochemical stimuli [1, 2]. These mediators, not only are involved in cartilage matrix degradation but also in cross-talk with synovial and subchondral bone cells.

Nowadays, curative treatments for OA remain missing and the management of OA focuses on the alleviation of symptoms. Current recommendations for the management of OA include a combination of non-pharmacological and pharmacological modalities. Moreover, for patients with severe OA, joint replacement is indicated [3]. Among non-pharmacological recommendations, exercise (land-based and water-based), biomechanical interventions, weight loss if overweight or obesity, and thermal modalities are widely recommended [3–5]. The most recommended pharmacological treatments include acetaminophen/paracetamol and non-steroidal anti-inflammatory drugs (NSAIDs) (topical or oral). Intra-articular corticosteroids are generally recommended for hip and knee OA [3–5]. However, the long-term use of NSAIDs and acetaminophen may be associated with detrimental effects, especially gastrointestinal adverse effects [6].

In this context, safer alternative treatments are needed. Such treatments could come from nutrition and more particularly from nutraceuticals. Some positive beneficial effects are highlighted with nutraceuticals in the course of OA [7, 8]. Among them, curcumin, which is the major component of turmeric, a yellow spice derived from the roots of the plant *Curcuma longa*. Anti-catabolic, anti-apoptotic and anti-inflammatory effects of curcumin are largely described *in vitro*. It is demonstrated that curcumin decreases nitric oxide (NO), prostaglandin E_2_ (PGE_2_), IL-6, IL-8, cyclooxygenase2 (COX2), inducible nitric oxide synthase (iNOS), MMP-3 and MMP-9 synthesis through the inhibition of nuclear factor κB (NF-κB) translocation and tumor necrosis factor (TNF)-α signaling pathways in chondrocytes [9–13]. Hydrolyzed collagen is obtained by the enzymatic hydrolysis of collagenous tissues, like for example, animal bone, and is generally recognized as a safe food ingredient by regulatory agencies [14, 15]. The main characteristic of hydrolyzed collagen is its amino acid composition, which is identical to collagen, thus providing high levels of glycine and proline, two amino acids essential for the stability and regeneration of cartilage [16, 17]. Proteoglycans synthesis, aggrecan gene expression and type II collagen synthesis are increased with hydrolyzed collagen in bovine and porcine articular chondrocytes [18, 19]. Epigallocatechin-3-gallate (EGCG) is a major component of the polyphenolic fraction of green tea and exhibits anti-oxidant, anti-tumor and anti-mutagenic activities [7, 20]. Advanced glycation end products (AGE)-induced expressions of TNF-α and MMP-13 are inhibited by EGCG in human OA chondrocytes. Otherwise, AGE-induced mitogen activated protein kinase (MAPK) signaling pathways and NF-κB activation are inhibited in that model [21]. The expression of mediators associated with OA pathogenesis is suppressed in human chondrocytes pre-treated with EGCG and then stimulated with IL-1β [22]. IL-1β induced mRNA expression and protein production of MMP-1 and MMP-13 are inhibited in an EGCG concentration dependant manner in human chondrocytes [23]. The transcription activity of NF-κB and the IL-1β-induced glycosaminoglycans (GAG) release from human cartilage explants are also reduced [23].

It has been reported that the majority of the pro-inflammatory and catabolic mediators linked to OA are regulated by NF-κB [24]. The subunits of NF-κB, p65 and p50, resides in the cytoplasm as an inactive complex in association with an inhibiting IκBα subunit. When phosphorylated, IκBα dissociated from the complex, and NF-κB, in response to phosphorylation of its subunit p65, is translocated to the nucleus, where it induces gene transcription and hence expression of more than 400 genes some of which are intimately involved in regulating apoptosis, proliferation and inflammation [25].

For the first time, this paper compares the effects of curcuminoids extract (C), hydrolyzed collagen (O) and green tea extract (T) in normal bovine and OA human chondrocytes cultured in monolayer. Further, this study investigates the synergic or additive effects of these compounds. Finally, we investigated the effects of these compounds on IL-1β induced NF-κB signaling pathway.

## Material and Methods

### Origin of chondrocytes and ethics statement

Normal bovine articular cartilage was obtained from “Public slaughterhouse of Liege” from the metacarpal/metatarsal-phalangeal joint of 1 to 2 years old steers shortly after death [11]. OA human articular cartilage was removed from knee joints of three patients undergoing total knee-replacement surgery [26]. All subjects provided written informed consent, and ethical approval (ethics committee agreement of Catholic University of Louvain, no. B403201111664) was granted for this study.

### Chondrocytes isolation

Full-depth articular cartilage was excised and immersed in Dulbecco’s Modified Eagle Medium (DMEM) (with phenol red and 4.5 g/L glucose) supplemented with N-(2-hydroxyethyl)piperazine-N’-(2-ethanesulfonic acid) (HEPES) 10 mM, penicillin (100 U/ml) and streptomycin (0.1 mg/ml) (all from Lonza, Verviers, Belgium). After three washings, chondrocytes were released from cartilage by sequential enzymatic digestions with 0.5 mg/ml hyaluronidase type IV S (Sigma-Aldrich, Bornem, Belgium) for 30 min at 37°C, 1 mg/ml pronase E (Merck, Leuven, Belgium) for 1 h at 37°C and 0.5 mg/ml clostridial collagenase IA (Sigma-Aldrich, Bornem, Belgium) for 16 to 20 h at 37°C. The enzymatically isolated cells were then filtered through a nylon mesh (70 μm), washed three times, counted and filled to the density of 0.25 x 10^6^ cells/ml (bovine chondrocytes) or 0.1 x 10^6^ cells/ml (human chondrocytes) of DMEM (with phenol red and 4.5 g/L glucose) supplemented with 10% fetal bovine serum, 10 mM HEPES, 100 U/ml penicillin, 0.1 mg/ml streptomycin, 2 mM glutamine (all from Lonza, Verviers, Belgium) and 20 μg/ml proline (Sigma-Aldrich, Bornem, Belgium)

### Chondrocytes culture

Cells were seeded in a 6-well plate at the density of 0.5 x 10^6^ cells/well (bovine chondrocytes) or 0.2 x 10^6^ cells/well (human chondrocytes) and cultured in monolayer for 5 days.

Chondrocytes were then cultured in monolayer until confluence (for about 24 hours) in DMEM (phenol red-free and containing only 1 g/L glucose) (Lonza, Verviers, Belgium) supplemented with 1% fetal bovine serum, 10 mM HEPES, 100 U/ml penicillin, 0.1 mg/ml streptomycin, 2 mM glutamine and 20 μg/ml proline. Only primary cultures were used to ensure the stability of chondrocyte phenotype. When normal bovine chondrocytes achieved confluence, the culture medium was removed and replaced by fresh culture medium containing curcuminoids extract (Naturex, Avignon, France) or hydrolyzed collagen (Gelita, Eberbach, Germany) or green tea extract (Naturex, Avignon, France) at the final concentration of 0.5 μg/ml, 2.5 μg/ml, 12.5 μg/ml or 62.5 μg/ml, with or without recombinant porcine IL-1β (10^-10^ M) (R&D System, Abingdon, UK). To investigate their potential synergic or additional effects, nutraceuticals were then tested alone at the final concentration of 12.5 μg/ml or in combination (12.5 μg/ml curcuminoids extract + 12.5 μg/ml hydrolyzed collagen; 12.5 μg/ml curcuminoids extract + 12.5 μg/ml green tea extract; 12.5 μg/ml curcuminoids extract + 12.5 μg/ml hydrolyzed collagen + 12.5 μg/ml green tea extract) in the absence or in the presence of recombinant porcine IL-1β (10^-10^ M). When OA human chondrocytes achieved confluence, the culture medium was removed and replaced by fresh culture medium and tested compounds alone or in combination (1–2–4 μg/ml curcuminoids extract + 1–2–4 μg/ml hydrolyzed collagen + 1–2–4 μg/ml green tea extract), at concentrations ranging between 1 to 4 μg/ml, and in the absence or in the presence of human IL-1β (10^-11^ M) (R&D System, Abingdon, UK). To investigate the effects of compounds on IL-1β induced NF-κB signaling pathway, OA human chondrocytes were pre-incubated with 4 μg/ml curcuminoids extract or with the combination 4 μg/ml curcuminoids extract + 4 μg/ml hydrolyzed collagen + 4 μg/ml green tea extract for 24 h and then co-treated with IL-1β (10^-11^ M) for 5 or 15 minutes or untreated. Curcuma extract is an oleoresin and is obtained from the roots of the plant *Curcuma longa*. Curcuminoids extract from Naturex is composed of natural extract and methylcellulose, and its content in curcuminoids is about 82% of which 75% are curcumin, 21% are demethoxycurcumin and 4% are bisdemethoxycurcumin. Hydrolyzed collagen from Gelita is a mix of different peptides. In average the peptides are composed by 30 amino acids, meaning a molecular weight of about 3 kDa. Glycine and proline represent more than 35% of total amino acids content. Green tea extract from Naturex, obtained from green tea leaves, contained natural extract and maltodextrin. Total polyphenols content is higher than 25%, catechins content higher than 12.5% and EGCG content higher than 9.3%. Curcuma extract was solubilized in tetrahydrofuran (Merck, Leuven, Belgium). Tetrahydrofuran was added in each condition to obtain final concentration at 0.1%. Hydrolyzed collagen and green tea extract were dissolved in water and filtered through a sterile mesh (0.20 μm). The effects of compounds were compared to controls consisting in same media without compounds and with or without IL-1β. Each culture condition was tested in triplicate in three independent chondrocytes cultures. Chondrocytes were incubated for 24 or 48 hours with the compounds and/or IL-1β. After 24 h of incubation, cells were scrapped, and RNA extraction was performed using RNeasy mini kit (Qiagen, Venlo, Netherlands) for quantitative real time polymerase chain reaction using the LightCycler 480 (Roche, Vilvoorde, Belgium).

After 48 h of incubation, conditioned culture media were collected for lactate dehydrogenase (LDH) release assay and then stored at-20°C until additional analysis. Cells were scrapped and homogenized in 500 μl of Tris-HCl buffer by ultrasonic dissociation for 20 s at 4°C, to measure desoxyribonucleic acid (DNA) content.

### Lactate dehydrogenase release assay

Cell viability was estimated by quantifying the release of LDH in the culture supernatant as previously described [12]. A sample of the supernatant or dilutions of standard solution (LDH from rabbit muscle) was mixed with Tris buffer (10 mM Tris-HCl (pH 8.5), 0.1% bovine serum albumin) containing 800 mM lactate. Then, colorimetric reagent, 1.6 mg/ml iodonitrotetrazolium chloride (Sigma-Aldrich, Bornem, Belgium), 4 mg/ml nicotinamide adenine dinucleotide (Roche Diagnostics, Brussels, Belgium), and 0.4 mg/ml phenazine methosulfate (Sigma-Aldrich, Bornem, Belgium), was added, and the absorbance at 492 nm was read after 10 min of incubation at room temperature.

### DNA assay

DNA content was measured in the cell extracts by a fluorimetric method using Hoechst [27].

### Quantitative real-time reverse transcriptase polymerase chain reaction (RT PCR)

RNA from bovine chondrocytes from 3 wells of each condition was isolated using RNeasy mini kit (Qiagen, Venlo, Netherlands). Then, RNA was reverse transcribed. Quantitative real time Polymerase Chain Reaction (qPCR) was performed by using the SYBR Premix Ex Taq (Tli RNaseH Plus) (Westburg, Leusden, Netherlands) as previously described [28]. The PCR template source was either first-strand complementary DNA (cDNA) (samples) or purified DNA standard (standard curve). Forward and reverse bovine primer sequences used to amplify the desired cDNA are presented in Table 1. Amplification was performed with a spectrofluorometric thermal cycler (LightCycler 480, Roche Diagnostics, Vilvoorde, Belgium). To standardize mRNA levels, we amplified hypoxanthine phosphoribosyltransferase (HPRT), a housekeeping gene, as an internal control. Gene expression was normalized by calculating the ratio between the number of cDNA copies of IL-6, COX2, iNOS, MMP-3, a disintegrin and metalloproteinase with thrombospondin motifs (ADAMTS) 4, and 5, and that of HPRT.

**Table 1:** Sequences of forward and reverse bovine primers used for PCR.

**Gene**	**Forward bovine primer**	**Reverse bovine primer**
**HPRT**	5’-AGTTTGGAAATACCTGGCG-3’	5’-AGTCTTTAGGCTCGTAGTGC-3’
**IL-6**	5’- TGGTGATGACTTCTGCTTTCC-3’	5’- TGCCAGTGTCTCCTTGC-3’
**COX2**	5’-GTCTGATGATGTATGCCACC-3’	5’-ACGTAGTCTTCAATCACAATCT-3’
**iNOS**	5’- GGCAAGCACCACATTGAGA-3’	5’- TGCGGCTGGATTTCGGA-3’
**MMP-3**	5’-TCTATGAAGGAGAAGCTGACATAAT-3’	5’-TTCATGGGCAGCAACAAG-3’
**ADAMTS 4**	5’- CTTTCAATGTCCCACAGGC-3’	5’- CAGGAACGGAAGCGGGTA-3’
**ADAMTS 5**	5’- GACACCCTGGGAATGGCA-3’	5’- CACAGAACTTGGAATCGTCA-3’

HPRT = hypoxanthine phosphoribosyltransferase, IL-6 = interleukin-6, COX2 = cyclooxygenase2, iNOS = inducible nitric oxide synthase, MMP-3 = matrix metalloproteinase-3, ADAMTS4 = a disintegrin and metalloproteinase with thrombospondin type I motifs 4, ADAMTS5 = a disintegrin and metalloproteinase with thrombospondin type I motifs 5.

### Nitrite assay

Nitric oxide (NO) production was determined by quantifying its derived product, nitrite, in the culture supernatant using a spectrophotometric method based upon the Griess reaction, as previously described [12]. Briefly, a sample of the supernatant or sodium nitrite (NaNO_2_) standard dilutions was mixed with Griess reagent (0.5% sulphanilamide, 0.05% naphtyl ethylene diamine dihydrochloride, 2.5% H_3_PO_4_). The absorption was measured at 540 nm.

### **Prostaglandin E**_2_ **assay**

PGE_2_ production was measured in the culture supernatant using the DetectX PGE_2_ High Sensitivity Immunoassay kit (Arbor Assays, Michigan, USA). Briefly, 100 μl of the supernatant or PGE_2_ standard dilutions were pipetted into a clear microtiter plate coated with an antibody to capture mouse IgG. A PGE_2_-peroxidase conjugate (25 μl) is added to the standards and supernatants in the wells. The binding reaction was initiated by the addition of 25 μl of a monoclonal antibody to PGE_2_. After an overnight incubation at 4°C, the plate was washed and 100 μl of substrate was added. After a short incubation, the reaction was stopped and the intensity of the generated colour was detected at 450 nm wavelength.

### Immunoassays for interleukin-6, matrix metalloproteinase-3 and aggrecans

IL-6, MMP-3, and aggrecans were measured by specific enzyme amplified sensitivity immunoassays (Invitrogen, Merelbeke, Belgium). IL-6 and MMP-3 productions were measured in culture supernatants, whereas aggrecans production was measured in culture supernatants and in the cell extracts.

### Western blotting

Cells were collected at 4°C and lysed on ice in 50 μl of buffer (25 mM Hepes, 150 mM NaCl, 0.5% Triton X-100, 10% glycerol, and 1 mM dithiothreitol) containing protease and phosphatase inhibitors (Roche, Vilvoorde, Belgium). After incubation at 4°C for 30 minutes, lysates were then centrifugated at 14,000 g for 30 minutes at 4°C to remove insoluble debris. Protein concentrations were determined using the BCA assay (Thermo Fisher Scientific, Erembodegem, Belgium). Total protein extracts (20 μg) were fractioned by electrophoresis on a polyacrylamide gel (10%) and transferred onto a PVDF membrane. Membranes were blocked for 1 h in TBS-Tween containing 5% nonfat dried milk (HSC70) or in 2% bovine serum albumin (phospho-NF-κB p65 and phospho-IκBα). Membranes were then incubated overnight at 4°C with primary antibodies. Anti-rabbit phospho-NF-κB p65 (1:1000 dilution), anti-rabbit phospho-IκBα (1:1000 dilution) and anti-rabbit HSC70 (1:1000 dilution) (Cell signaling, Boston, USA) were used. Horse-radish peroxidase (HRP)-linked anti-rabbit IgG antibody (1:2000 dilution) was used as secondary antibody (Cell signaling, Boston, USA). The reaction was revealed with the ECL Western blotting substrate (Thermo Fisher Scientific, Erembodegem, Belgium).

### Statistical analysis

Results were normalized to the HPRT gene expression or to the DNA content of the cells and expressed as the mean ± SEM or reported to the control and expressed in percent. Statistical significance is assessed using a parametric one-way ANOVA, followed by Dunnett’s multiple comparison post-test for statistical significance compared to control, and followed by Tukey’s multiple comparison post-test for statistical significance compared to the mixture. Differences were considered statistically significant at p-value < 0.05.

## Results

### Effects of curcuminoids extract, hydrolyzed collagen and green tea extract on normal bovine chondrocytes in monolayer

Bovine chondrocytes were cultured for 24 hours (gene expressions analysis) or 48 hours (protein productions analysis) in the absence or in the presence of IL-1β and with or without curcuminoids extract (C), hydrolyzed collagen (O) or green tea extract (T) added alone or in combination (COT). Cell viability, evaluated by LDH release, and DNA content, was not significantly modified by compounds alone or in combination, with or without IL-1β (data not shown).

In basal conditions, NO and PGE_2_ productions were undetectable. However, bovine chondrocytes expressed 0.43 ± 0.23 copies of IL-6 mRNA, 7.83 ± 2.83 copies of iNOS mRNA, 1.62 ± 0.93 copies of COX2 mRNA, 3.00 ± 1.00 copies of MMP-3 mRNA, 0.6 ± 0.4 copies of ADAMTS4 mRNA, 13.13 ± 2.64 copies of ADAMTS5 mRNA and 146.9 ± 82.51 copies of aggrecans mRNA per copy of HPRT. The compounds did not significantly affect the basal expression of these genes.

**Fig 1:**
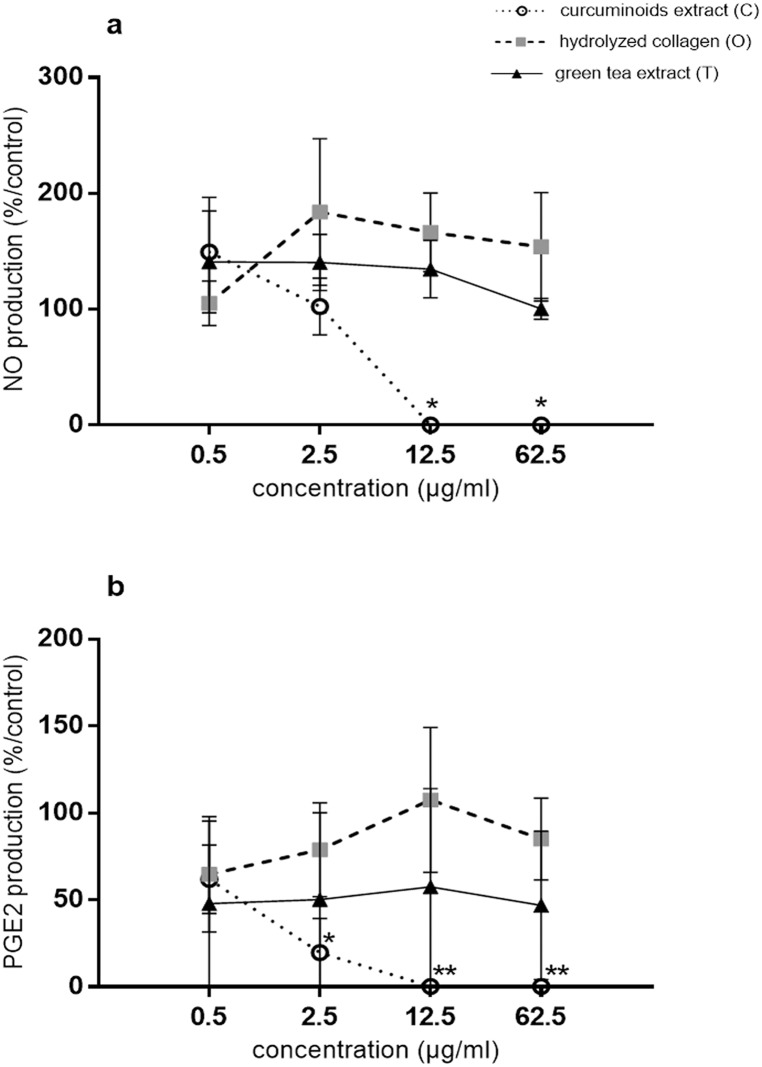
Compounds effects on IL-1β stimulated bovine chondrocytes in monolayer (production). Results were expressed as percent of IL-1β treated chondrocytes culture (control). *p<0.05 versus control, **p<0.01 versus control. (a) NO = nitric oxide, (b) PGE_2_ = prostaglandin E_2_.

In the presence of IL-1β, the productions of NO and PGE_2_ were 5.16 ± 1.42 nmol/μg DNA and 8,372.95 ± 5,187.35 pg/μg DNA, respectively. IL-1β increased the expression of the genes coding for IL-6, iNOS, COX2, MMP-3, ADAMTS4, ADAMTS5 by 178.16 ± 8.04, 17.87 ± 7.72, 11.18 ± 2.48, 88.41 ± 45.19, 25.67 ± 14.73, 3.26 ± 1.03-fold, respectively and decreased aggrecans by 7.00 ± 3.32-fold. In the presence of IL-1β and at the concentrations of 12.5 and 62.5 μg/ml, C significantly and fully inhibited NO production (p = 0.0101), while O and T were without effects (Fig. 1a). At the concentrations of 2.5, 12.5 and 62.5 μg/ml, C significantly inhibited IL-1β stimulated PGE_2_ production (IL-1β: 8,372.95 ± 5,187.35 pg/μg DNA; 2.5 μg/ml: 1,753.93 ± 878.64 pg/μg DNA, p = 0.0478; 12.5 μg/ml: 9.19 ± 9.19 pg/μg DNA, p = 0.0076 and 62.5 μg/ml: 9.31 ± 9.31 pg/μg DNA, p = 0.0076) (Fig. 1b). In contrast, O and T had no significant effect on PGE_2_ production.

**Fig 2:**
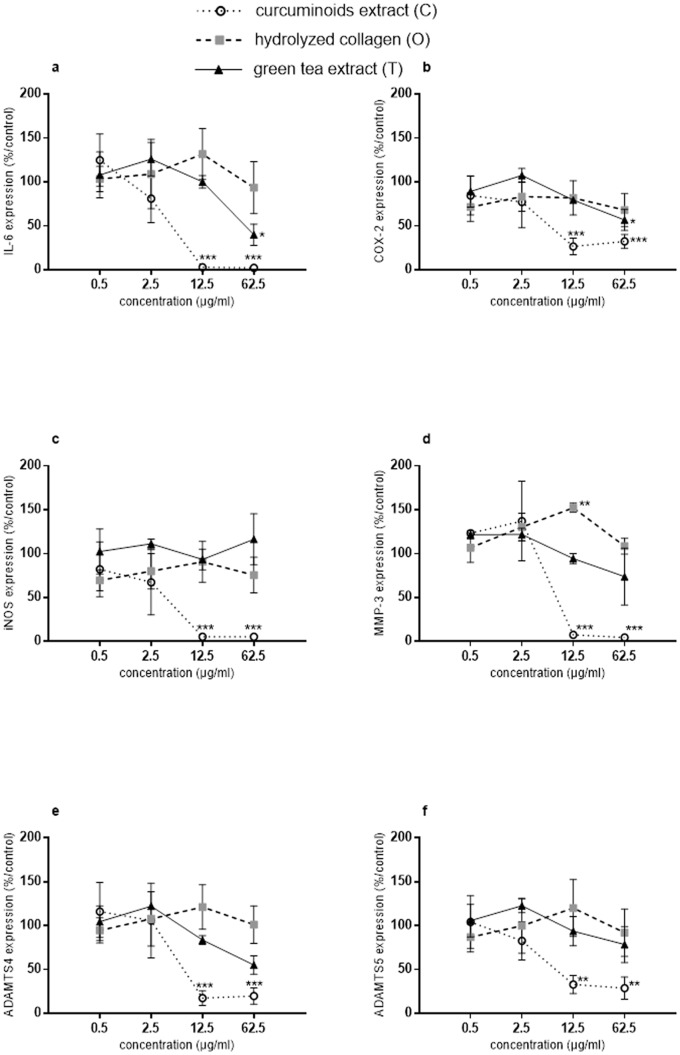
Compounds effects on IL-1β stimulated bovine chondrocytes in monolayer (expression). Results were expressed as percent of IL-1β treated chondrocytes culture (control). *p<0.05 versus control, **p<0.01 versus control, ***p<0.001 versus control. (a) IL-6 = interleukin-6, (b) COX2 = cyclooxygenase2, (c) iNOS = inducible nitric oxide synthase, (d) MMP-3 = matrix metalloproteinase-3, (e) ADAMTS4 = a disintegrin and metalloproteinase with thrombospondin type 1 motifs 4 and (f) ADAMTS5 = a disintegrin and metalloproteinase with thrombospondin type 1 motifs 5.

In the presence of IL-1β and at the concentrations of 12.5 and 62.5 μg/ml, C significantly inhibited IL-6 (IL-1β: 100.23 ± 18.99 copies mRNa per copy of HPRT; 12.5 μg/ml: 3.67 ± 2.52 copies mRNA per copy of HPRT, p<0.0001; 62.5 μg/ml: 2.37 ± 1.28 copies mRNA per copy of HPRT, p<0.0001), COX2 (IL-1β: 13.93 ± 4.52 copies mRNA per copy of HPRT; 12.5 μg/ml: 4.79 ± 2.77 copies mRNA per copy of HPRT, p<0.0001; 62.5 μg/ml: 5.01 ± 2.9 copies mRNA per copy of HPRT, p = 0.0001) iNOS (IL-1β: 99.43 ± 1.59 copies mRNA per copy of HPRT; 12.5 μg/ml: 5.33 ± 3.26 copies mRNA per copy of HPRT, p<0.0001; 62.5 μg/ml: 5.33 ± 1.41 copies mRNA per copy of HPRT p<0.0001), MMP-3 (IL-1β: 220.05 ± 38.5 copies mRNA per copy of HPRT; 12.5 μg/ml: 18.25 ± 8.86 copies mRNA per copy of HPRT, p<0.0001; 62.5 μg/ml: 9.1 ± 2.2 copies mRNA per copy of HPRT, p<0.0001), ADAMTS4 (IL-1β: 7.53 ± 2.24 copies mRNA per copy of HPRT; 12.5 μg/ml: 1.47 ± 0.75 copies mRNA per copy of HPRT, p = 0.0005; 62.5 μg/ml: 1.5 ± 0.85 copies mRNA per copy of HPRT, p = 0.0008) and ADAMTS5 (IL-1β: 37.67 ± 4.39 copies mRNA per copy of HPRT; 12.5 μg/ml: 11.87 ± 3.42 copies mRNA per copy of HPRT, p = 0.0073; 62.5 μg/ml: 9.77 ± 3.01 copies mRNA per copy of HPRT, p = 0.0037) gene expressions (Fig. 2a, b, c, d, e, f). In the presence of IL-1β and at the concentration of 62.5 μg/ml, T significantly inhibited IL-6 (IL-1β: 100.23 ± 18.99 copies mRNA per copy of HPRT; 62.5 μg/ml: 35.6 ± 4.92 copies mRNA per copy of HPRT, p = 0.0309) and COX2 (IL-1β: 13.93 ± 4.52 copies mRNA per copy of HPRT; 62.5 μg/ml: 9 ± 4.69 copies mRNA per copy of HPRT, p = 0.0311) gene expressions (Fig. 2a, b). In the presence of IL-1β and at the concentration of 12.5 μg/ml, O significantly increased MMP-3 gene expression (IL-1β: 220.05 ± 38.5 copies mRNA per copy of HPRT; 12.5 μg/ml: 337.35 ± 67.81 copies mRNA per copy of HPRT, p = 0.0063) (Fig. 2d).

We have also tested two compounds in combination, CO and CT. CO and CT tended to be more efficient than C on all parameters. The differences between C and CO groups were not statistically significant. In contrast, CT reduced higher IL-1β-stimulated MMP-3 expression than C (p = 0.0386) (Table 2).

**Table 2:** Compounds effects on IL-1β stimulated bovine chondrocytes in monolayer.

			C	CO	CT
Production	NO	% of ctrl	3,84	0,000001	0,000001
		p-value vs ctrl	**<0,0001**	**<0,0001**	**<0,0001**
		p-value vs C		0,9996	0,9996
	PGE2	% of ctrl	0,9323	0,4982	0,000001
		p-value vs ctrl	0,9925	0,9924	0,9922
		p-value vs C		>0,9999	>0,9999
Gene expression	IL-6	% of ctrl	16,37	10,93	0,47
		p-value vs ctrl	**<0,0001**	**<0,0001**	**<0,0001**
		p-value vs C		0,9988	0,7944
	COX2	% of ctrl	48,72	42,36	13,28
		p-value vs ctrl	0,0719	**0,0385**	**0,0021**
		p-value vs C		0,9998	0,524
	iNOS	% of ctrl	14,18	8,671	3,757
		p-value vs ctrl	**<0,0001**	**<0,0001**	**<0,0001**
		p-value vs C		0,9879	0,8005
	MMP-3	% of ctrl	42,5	15,41	1,21
		p-value vs ctrl	**0,0011**	**<0,0001**	**<0,0001**
		p-value vs C		0,2934	**0,0386**
	ADAMTS4	% of ctrl	45,28	31,81	17,16
		p-value vs ctrl	**0,0279**	**0,0061**	**0,0012**
		p-value vs C		0,9818	0,6516
	ADAMTS5	% of ctrl	53,33	48,02	24,32
		p-value vs ctrl	0,1905	0,1261	**0,0168**
		p-value vs C		>0,9999	0,8233

Ctrl = IL-1β stimulated bovine chondrocytes, C = curcuminoids extract, CO = curcuminoids extract + hydrolyzed collagen, CT = curcuminoids extract + green tea extract, NO = nitric oxide, PGE_2_ = prostaglandin E_2_, IL-6 = interleukin-6, COX2 = cyclooxygenase-2, iNOS = inducible nitric oxide synthase, MMP-3 = matrix metalloproteinase-3, ADAMTS4 = a disintegrin and metalloproteinase with thrombospondin type I motifs 4, ADAMTS5 = a disintegrin and metalloproteinase with thrombospondin type I motifs 5.

**Fig 3:**
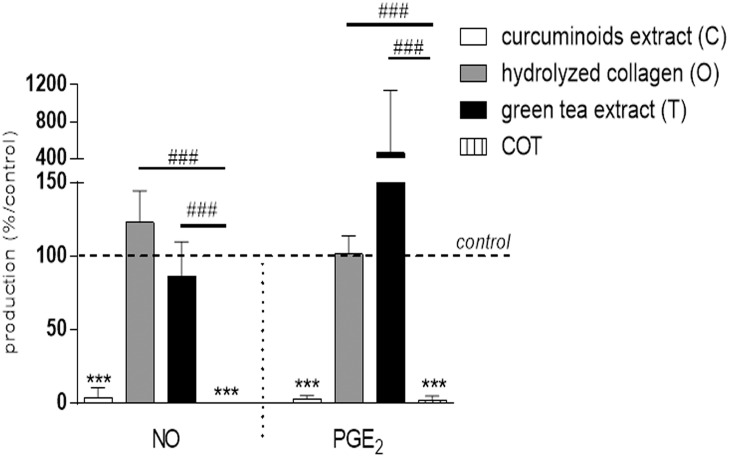
Compounds effects, separately or in combination, on IL-1β stimulated bovine chondrocytes in monolayer (production). Results were expressed as percent of IL-1β treated chondrocytes culture (control). ***p<0.001 versus control, ###p<0.001 versus COT. NO = nitric oxide, PGE_2_ = prostaglandin E_2_.

**Fig 4:**
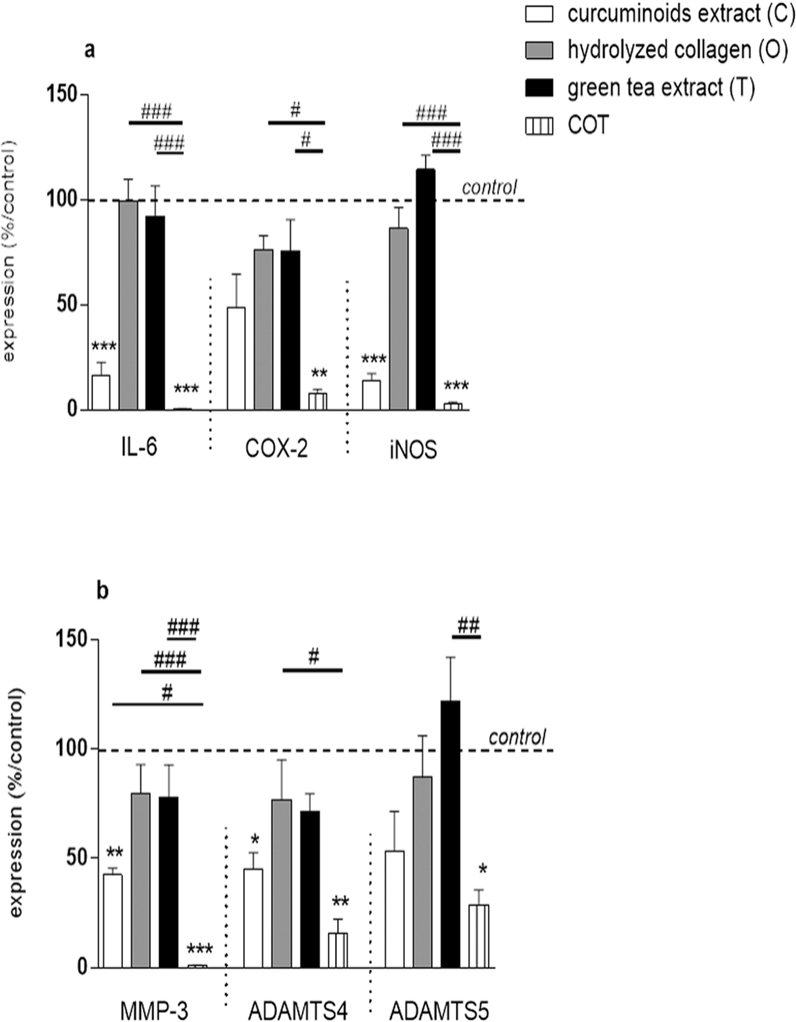
Compounds effects, separately or in combination, on IL-1β stimulated bovine chondrocytes in monolayer (expression). Results were expressed as percent of IL-1β treated chondrocytes culture (control). *p<0.05 versus control, **p<0.01 versus control, ***p<0.001 versus control, #p<0.05 versus COT, ##p<0.01 versus COT, ###p<0.001 versus COT. (a) IL-6 = interleukin-6, COX2 = cyclooxygenase2, iNOS = inducible nitric oxide synthase, (b) MMP-3 = matrix metalloproteinase-3, ADAMTS4 = a disintegrin and metalloproteinase with thrombospondin type 1 motifs 4 and ADAMTS5 = a disintegrin and metalloproteinase with thrombospondin type 1 motifs 5.

The mixture COT significantly inhibited IL-1β stimulated NO and PGE_2_ productions (p<0.0001), but the inhibitory effect was not higher than C alone (Fig. 3). The magnitude of COT inhibitory effect was higher than C alone on IL-1β-stimulated IL-6 mRNA (52.73 ± 15.64 copies mRNA per copy of HPRT for C vs 5.07 ± 4.32 copies mRNA per copy of HPRT for COT, p<0.0001), COX2 mRNA (6.2 ± 2.04 copies mRNA per copy of HPRT for C vs 1.07 ± 0.38 copies mRNA per copy of HPRT for COT, p = 0.0035), iNOS mRNA (11.27 ± 5.04 copies mRNA per copy of HPRT for C vs 3.5 ± 2.41 copies mRNA per copy of HPRT for COT, p<0.0001) (Fig. 4a), MMP-3 mRNA (85.8 ± 44.88 copies mRNA per copy of HPRT for C vs 1.87 ± 0.93 copies mRNA per copy of HPRT for COT, p<0.0001), ADAMTS4 mRNA (4.47 ± 2.09 copies mRNA per copy of HPRT for C vs 1.23 ± 0.48 copies mRNA per copy of HPRT for COT, p = 0.003) and ADAMTS5 mRNA (50.57 ± 1.62 copies mRNA per copy of HPRT for C vs 29.67 ± 4.68 copies mRNA per copy of HPRT for COT, p = 0.0248) (Fig. 4b) gene expressions.

### Effects of curcuminoids, hydrolyzed collagen and green tea extract on OA human chondrocytes in monolayer

Primary OA human chondrocytes were cultured in monolayer until confluence and then incubated for 48 hours in the absence or in the presence of IL-1β and with or without C, O or T added alone or in combination (COT). Cell viability, evaluated by LDH release, and DNA content, was not affected by these compounds added alone or in combination, with or without IL-1β (data not shown).

**Fig 5:**
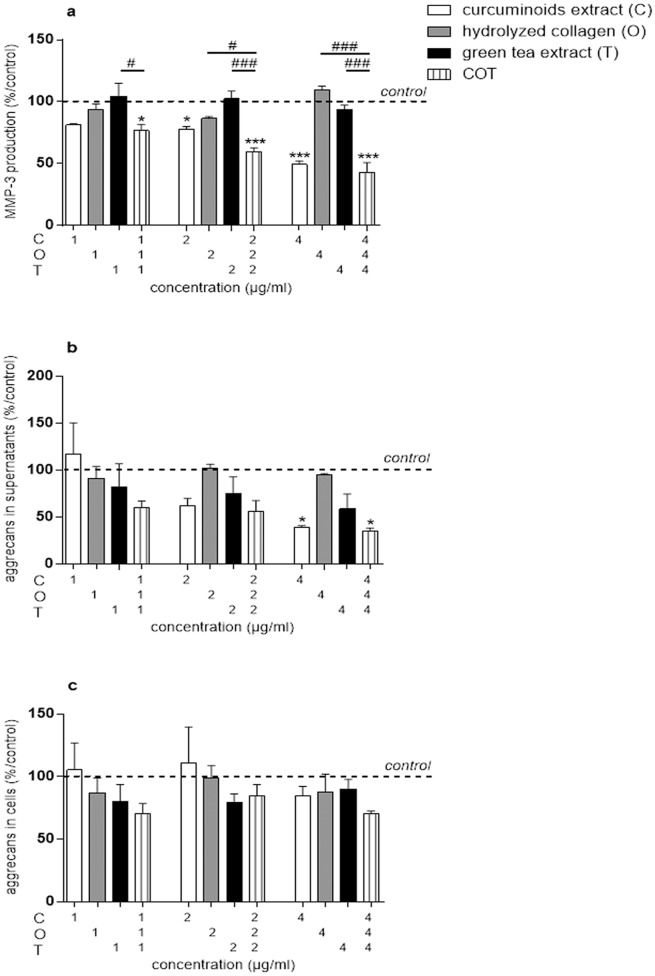
Compounds effects, separately or in combination, on human chondrocytes in monolayer (production). Results were expressed as percent of control. *p<0.05 versus control, ***p<0.001 versus control, #p<0.05 versus COT, ###p<0.001 versus COT. (a) MMP-3 = matrix metalloproteinase-3, (b, c) aggrecans.

In basal conditions, NO production was undetectable while the content of IL-6, MMP-3 and aggrecans in culture supernatants were 232.32 ± 85.3 pg/μg DNA, 207.34 ± 15.83 ng/μg DNA and 538 ± 86.48 ng/μg DNA, respectively. The aggrecans content in chondrocytes extract was 28.92 ± 4.61 ng/μg DNA. In basal conditions, MMP-3 production was significantly inhibited by C at 2 (151.86 ± 19.03 ng/μg DNA, p = 0.0166) and 4 μg/ml (92.84 ± 19.49 ng/μg DNA, p<0.0001) (Fig. 5a). At the concentration of 4 μg/ml, C significantly decreased the aggrecans content in culture supernatants (214.87 ± 41.34 ng/μg DNA, p = 0.0232) (Fig. 5b), but had no effect on aggrecans content in chondrocytes extract (Fig. 5c). None of the compounds, neither the combination, showed significant effects on NO and IL-6 productions in the absence of IL-1β on human chondrocytes (data not shown). O and T had no significant effect on human chondrocytes in basal conditions.

In basal conditions, MMP-3 production was significantly inhibited by the COT mixture at 1:1:1 (139.1 ± 30.13 ng/μg DNA, p = 0.0105), 2:2:2 (110.95 ± 20.97 ng/μg DNA, p<0.0001) and 4:4:4 μg/ml (78.03 ± 14.85 ng/μg DNA, p<0.0001) (Fig. 5a). At the concentration of 1:1:1 μg/ml, COT inhibited MMP-3 production while C, O or T individually had no effect. This indicated that, at the concentration of 1 μg/ml, C, O and T acted in synergy on this parameter. Further, COT at 4:4:4 μg/ml significantly decreased the aggrecans content in culture supernatants (192.91 ± 45.98 ng/μg DNA, p = 0.0133) (Fig. 5b), but had no significant effect on aggrecans content in chondrocytes extract (Fig. 5c). However, COT had a similar effect on the aggrecans in culture supernatants than C.

**Fig 6:**
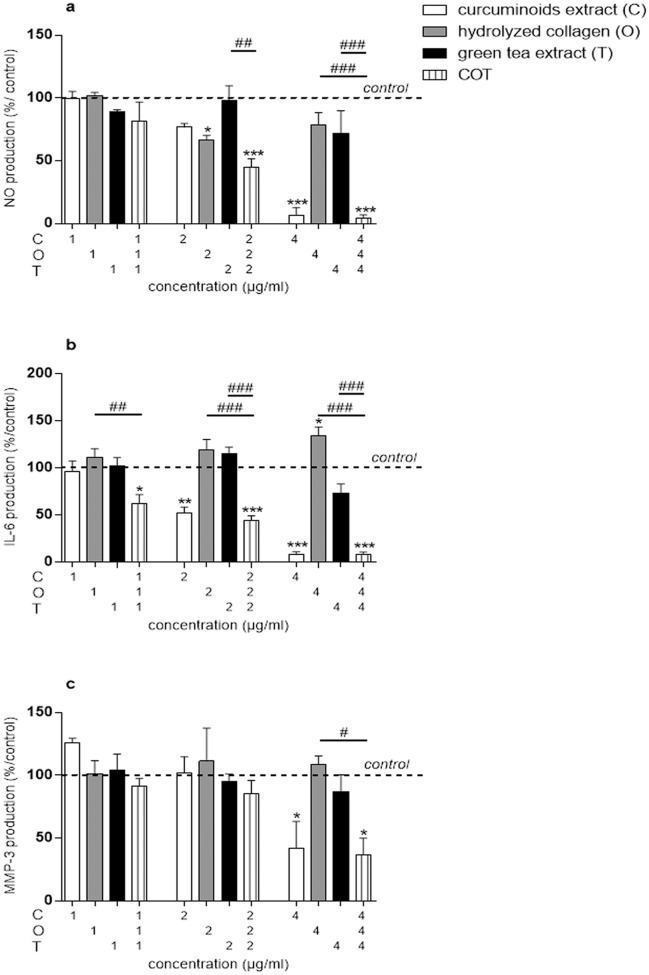
Compounds effects, separately or in combination, on IL-1β stimulated human chondrocytes in monolayer (production). Results were expressed as percent of IL-1β treated chondrocytes culture (control). *p<0.05 versus control, **p<0.01 versus control, ***p<0.001 versus control, #p<0.05 versus COT, ##p<0.01 versus COT, ###p<0.001 versus COT. (a) NO = nitric oxide, (b) IL-6 = interleukin-6, (c) MMP-3 = matrix metalloproteinase-3.

In the presence of 10^-11^ M IL-1β, the production of NO was 37.92 ± 6.04 nmol/μg DNA. IL-1β significantly increased the production of IL-6 and MMP-3 by 2,114.91 ± 789.59 and 13.61 ± 2.7-fold, respectively. In contrast, IL-1β inhibited aggrecans content in cells extract and supernatants by 1.37 ± 0.36 and 4.72 ± 1.83-fold, respectively. C significantly inhibited IL-1β stimulated NO production at the concentration of 4 μg/ml (IL-1β: 37.92 ± 6.04 nmol/μg DNA; 4 μg/ml: 2.75 ± 2.75 nmol/μg DNA, p<0.0001) (Fig. 6a), IL-1β stimulated IL-6 production at the concentrations of 2 (IL-1β: 374,153.98 ± 55,870.58 pg/μg DNA; 2 μg/ml: 198,865.78 ± 51,036.33 pg/μg DNA, p = 0.0029) and 4 μg/ml (IL-1β: 374,153.98 ± 55,870.58 pg/μg DNA; 4 μg/ml: 26,185.84 ± 9,712.2 pg/μg DNA, p<0.0001) (Fig. 6b) and IL-1β stimulated MMP-3 production at the concentration of 4 μg/ml (IL-1β: 2,839.88 ± 661.96 ng/μg DNA; 4 μg/ml: 946.97 ± 254.48 ng/μg DNA, p = 0.024) (Fig. 6c). O significantly inhibited IL-1β stimulated NO production at the concentration of 2 μg/ml (IL-1β: 37.92 ± 6.04 nmol/μg DNA; 2 μg/ml: 24.87 ± 2.96 nmol/μg DNA, p = 0.0394) (Fig. 6a) but had no significant effect on the other parameters. T alone had no significant effect on IL-1β treated human chondrocytes.

The COT mixture significantly decreased IL-1β-stimulated NO production at the concentrations of 2:2:2 (IL-1β: 37.92 ± 6.04 nmol/μg DNA; 2:2:2 μg/ml: 17.74 ± 4.72 nmol/μg DNA, p = 0.0003) and 4:4:4 μg/ml (IL-1β: 37.92 ± 6.04 nmol/μg DNA; 4:4:4 μg/ml: 1.65 ± 1.15 nmol/μg DNA, p<0.0001) (Fig. 6a). At the concentration of 4:4:4 μg/ml, COT and C effects on NO were not significantly different whereas, at the concentration of 2:2:2 μg/ml, COT had a higher inhibitory effect than O (Fig. 6a). The COT mixture significantly decreased IL-1β stimulated IL-6 production at the concentrations of 1:1:1 (IL-1β: 374,153.98 ± 55,870.58 pg/μg DNA; 1:1:1 μg/ml: 238,658.42 ± 61,332.79 pg/μg DNA, p = 0.0259), 2:2:2 (IL-1β: 374,153.98 ± 55,870.58 pg/μg DNA; 2:2:2 μg/ml: 165,110.45 ± 22,791.39 pg/μg DNA, p = 0.0006) and 4:4:4 μg/ml (IL-1β: 374,153.98 ± 55,870.58 pg/μg DNA; 4:4:4 μg/ml: 28,127.75 ± 6,569.56 pg/μg DNA, p<0.0001) (Fig. 6b). C, O and T added separately at the concentration of 1 μg/ml had no effect on IL-6 production. In contrast, COT mixture (1:1:1 μg/ml) significantly reduced IL-1β stimulated IL-6 production (p = 0.0259). However, at the concentrations of 2:2:2 and 4:4:4 μg/ml, there was no additive effect of COT on IL-1β stimulated IL-6 production, compared to C (Fig. 6b). The COT mixture, at 4:4:4 μg/ml, decreased IL-1β stimulated MMP-3 production (IL-1β: 2,839.88 ± 661.96 ng/μg DNA; 4:4:4 μg/ml: 863.56 ± 181.07 ng/μg DNA, p = 0.0119) but this inhibitory effect was similar to that of C (Fig. 6c).

**Fig 7:**
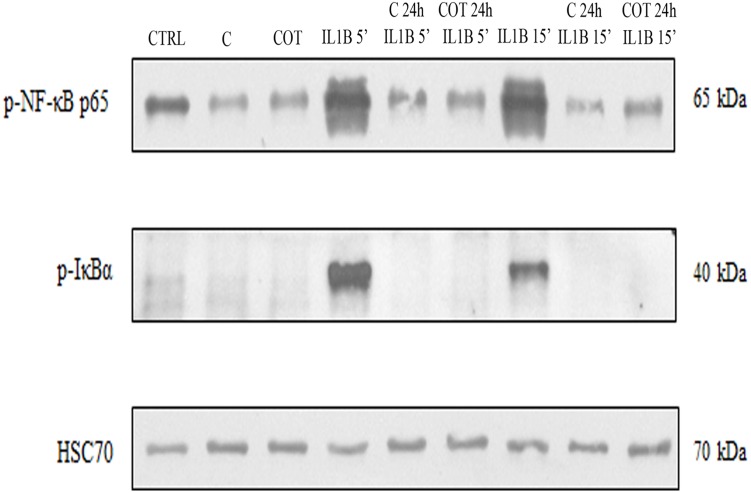
Compounds effects on IL-1β induced phosphorylations of NF-κB p65 and IκBα subunits. Western blot analysis with IL-1β stimulated chondrocytes total protein extracts. Chondrocytes were pre-incubated with C or COT for 24 h and then co-treated with IL-1β (10^-11^ M) for 5 or 15 min. The protein extracts were probed for phospho NF-κB p65, phospho IκBα and HSC70 (control) by Western blot analysis using specific antibodies.

We next investigated the effects of C and COT on human chondrocytes on IL-1β induced NF-κB activation. Protein extracts of chondrocytes were probed for the phosphorylated p65 NF-κB subunit after pre-treatment with C or COT for 24 h followed by co-treatment or not with IL-1β (10^-11^ M) for 5 or 15 minutes. C and COT inhibited IL-1β induced phosphorylation and translocation of p65 in nuclear extracts of chondrocytes (Fig. 7). Further, to examine whether inhibition of IL-1β induced NF-κB activation occurred through inhibition of IκBα degradation, protein extracts of chondrocytes were probed for the phosphorylated IκBα subunit after pre-treatment with C or COT for 24 h followed by co-treatment or not with IL-1β (10^-11^ M) for 5 or 15 minutes. IL-1β induced IκBα degradation as early as 5 min, but IL-1β could not induce IκBα subunit degradation in C and COT pre-treated chondrocytes (Fig. 7).

## Discussion

This study aimed 1) to compare the effects of three nutraceuticals, curcuminoids extract, hydrolyzed collagen and green tea extract on chondrocytes metabolism, 2) to investigate a potential synergic effect between these compounds and 3) to examine whether the effects of curcuminoids extract and the combination of the three compounds on human chondrocytes involved the NF-κB signaling pathway. Numerous mediators contribute to both degradative and nociceptive pathways associated with the progression of OA [29]. Among these mediators, some cytokines like IL-1β play a key role in OA, by stimulating cartilage degradation and by triggering synovial membrane inflammation [30]. *In vitro*, IL-1β strongly stimulates cartilage degradation, and inhibits proteoglycan and collagen synthesis [2, 31–33]. IL-1β increases the production of a large spectrum of proteolytic enzymes such as ADAMTS4, ADAMTS5, MMP-3 and a variety of other cytokines, including IL-6 and IL-8, by chondrocytes. For these reasons, we treated human primary chondrocytes with IL-1β to mimic OA chondrocyte metabolic responses. Normalizing chondrocyte metabolism and, more particularly, counteracting the deleterious effects of IL-1β, are targets for pharmaceutical agents in OA. Evidence to date indicates that in addition to IL-1β, IL-6 and IL-8 can also promote articular cartilage extracellular matrix degradation or synergize with other cytokines to amplify and accelerate cartilage destruction [29, 33]. As expected, IL-1β stimulation of chondrocytes significantly induced the expression of COX2, iNOS, ADAMTS4 and ADAMTS5 and the production of NO and PGE_2_ in our experiments. Moreover, IL-1β treatment significantly induced the mRNA expression as well as protein production of IL-6 and MMP-3, and significantly reduced the mRNA expression as well as protein production of aggrecans by chondrocytes.

We have demonstrated that C had beneficial effects on chondrocytes. In fact, C counteracted the deleterious effects of IL-1β on chondrocytes. More precisely, C reduced the stimulating effect of IL-1β on pro-inflammatory and pro-catabolic mediators like cytokines, NO or MMP. These effects were mediated by inhibiting NF-kB signaling pathway. C inhibited IL-1β induced activation of NF-κB and its translocation to the chondrocyte nucleus. Further, C abolished IL-1β induced degradation of IκBα subunit. Our results were consistent with previously published results showing that curcumin suppressed IL-1β induced NF-κB activation via inhibition of IκBα phosphorylation and p65 phosphorylation [10], and prevented nuclear translocation of NF-κB [9].

These data confirm those of previous studies demonstrating that purified curcumin inhibited IL-1β stimulated NO, PGE_2_ and MMP-3 productions, and IL-1β stimulated IL-6 and IL-8 gene expressions in primary bovine normal chondrocytes and human OA chondrocytes [11, 12], by inhibiting the phosphorylation of Iκβα [34]. In addition, we demonstrated that C significantly inhibited gene expressions of ADAMTS4 and ADAMTS5, two enzymes directly involved in aggrecans cleavage. We also observed that C reduced aggrecan content in the culture supernatant without affecting significantly aggrecan content in cells extract. This decrease of aggrecan in culture supernatant could result of a reduction of aggrecan synthesis or of an increase of aggrecan degradation as antibodies used in the aggrecan immunoassay target the hyaluronic acid binding region or keratan sulfates residues. Therefore, an increase of aggrecan degradation reduces the amount of aggrecan measured in culture supernatant. However, we failed to show that C increased ADAMTS gene expression or reduced aggrecan gene expression. The monolayer culture model is not the best model to investigate aggrecan production and accumulation in the extracellular matrix. To verify our hypothesis, it would be interesting to test C in chondrocytes cultured in alginate beads. More surprisingly were the few effects of T and O. T significantly decreased IL-6 and COX-2 gene expressions but had no significant effects on the other parameters while O had no significant effect. This observation contrasts with the results of other studies suggesting that O and T could have beneficial effects on cartilage. Indeed, it was demonstrated that Proline-Hydroxyproline (Pro-Hyp), the major hydroxyproline-containing peptide in human blood after oral ingestion of collagen hydrolysates [17], increased the aggrecan mRNA levels in murine chondrocytes [35] and collagen hydrolysates and Pro-Hyp inhibited murine chondrocyte differentiation into mineralized chondrocytes and increased glycosaminoglycans production [35]. EGCG, the most abundant catechin in green tea, suppressed the inflammatory response in human chondrocytes [22]. Other data proved that EGCG decreased the production of TNFα [21]. These discrepancies can be explained by the cell type. Indeed, the beneficial effects of hydrolyzed collagen were observed on a murine chondrocytic cell line whereas our research was performed on primary bovine and human chondrocytes. Moreover, the effects of hydrolyzed collagen may differ according the origin and formulation of collagen hydrolysates [15]. Furthermore, to show a decrease in inflammatory response, primary human chondrocytes were pre-treated with different doses of EGCG for 1 or 2 hours prior to stimulation (with advanced glycation end products [21] or with 5000 pg/ml IL-1β) while we co-treated our primary human chondrocytes with T and with IL-1β at a lower concentration (170 pg/ml = 10^-11^ M) for 48 hours. It was already shown that EGCG individually marginally inhibit COX-2 expression and PGE_2_ production in cytokine-activated equine chondrocytes. Finally, EGCG, in combination with avocado and soya unsaponifiables, reduced COX2 expression close to non-activated control levels and significantly inhibit PGE_2_ production [36].

For the first time, this paper described the effects of C, O and T in combination on chondrocyte metabolism. In bovine chondrocytes, the three compounds showed additive inhibitory effects on IL-1β stimulated IL-6, iNOS and ADAMTS4 gene expressions. Moreover, the three compounds acted synergically to inhibit IL-1β stimulated COX2, MMP-3 and ADAMTS5 gene expressions. COT showed a higher effect on these parameters than C alone. Further, the inhibitory effect of COT on IL-1β-induced COX-2, iNOS, MMP-3 and ADAMTS4 expressions tended to be greater than that of CO or CT. In human chondrocytes, COT had an additive inhibitory effect on MMP-3 and IL-1β stimulated NO production and acted synergically on IL-1β stimulated IL-6 production.

Altogether, these data gave a rationale for combining these compounds in the management of OA.

One major limitation of our study is that compounds have been tested on a limited number of cartilage specimens. Additional experiments are needed, for example in order to reach the significance of the effects and identify the best responders among a large panel of specimens.

Of course, before recommending these natural products in the management of OA, some clinical trials are required to demonstrate the beneficial effects of these nutraceuticals on OA symptoms and structural changes. Another major concern is the absence of information about dietary supplements bioavailability. This point is particularly true for polyphenol like curcumin. In fact, natural curcumin is known for its very low bioavailability. This point is crucial to envisage its administration by oral route. For example, the average peak serum concentrations after oral intake of 4, 6 and 8 g of curcumin per day were 0.51 ± 0.11, 0.63 ± 0.06 and 1.77 ± 1.87 μM, respectively [37]. The serum concentration of curcumin peaked at 1–2 h after oral intake of curcumin and gradually declined within 12 hours. Urinary excretion of curcumin was undetectable [37]. This mean that the concentrations of curcuminoids extract tested in this *in vitro* study (4 μg/ml _~_ 10 μM) are superior to those found in plasma after oral administration of high doses of natural curcumin. Therefore, the extrapolation of our *in vitro* data to human nutrition must be done with caution. However, many effort have been made to increase curcumin bioavaibility. Recently, we performed a Phase I pharmacokinetics study on Flexofytol, a high bioavailable turmeric extract with a water solubility increased 4000 times, that was run on 2 groups of 12 healthy individuals. Each group received orally 1 (42 mg curcumin) or 2 capsules (84 mg of curcumin) of Flexofytol respectively. With 2 capsules administered orally, the mean of Cmax on 12 individuals was 0.9 μM, with a statistical extrapolation at 1.6 μM with 4 capsules (administering 84 mg and 168 mg of curcumin respectively). These values are closer with those used in our *in vitro* study.

The challenge is to develop a curcumin with an increased bioavailability, with the aim of reaching plasma concentrations that have demonstrated biological activity. In this purpose, curcumin has been dissolved in oil and then absorbed into chylomicrons, to propose a surface-controlled water-dispersible curcumin, with no modification of its structure [38, 39]. Curcumin was also co-administered with piperine, an inhibitor of hepatic and intestinal glucuronidation [40] or included in a phosphatidylcholine phytosome complex [41, 42]. The clinical relevance of these new formulations compared to the native curcumin should be demonstrated in good clinical trials.

Altogether, these *in vitro* results indicate that the mixture COT might reduce inflammation and pain in OA by reducing the synthesis of inflammatory and catabolic mediators by chondrocytes, probably by inhibiting NF-κB activation. These findings provide a preclinical basis for the *in vivo* testing of the combinations and suggest that these natural compounds could be helpful to alleviate symptoms in OA patients. These results support firstly the development and use of the mixture COT as an anti-inflammatory / anti-osteoarthritic agent for treatment of OA.
